# Co-occurrence of Wilsons disease and systemic lupus erythematosus: a case report and literature review

**DOI:** 10.1186/s12876-021-01814-5

**Published:** 2021-05-21

**Authors:** Lishan Xu, Bin Liu, Zhaoyang Liu, Ning Tang, Chunhui She, Jing Wang, Bo Zang, Yifei Yang

**Affiliations:** Department of Rheumatology, The Affiliated Hospital of Qingdao University, Qingdao, 260071 China

**Keywords:** Systemic lupus erythematosus, Liver fibrosis, Wilsons disease, Diagnosis

## Abstract

**Background:**

Wilsons disease (WD) is a rare autosomal recessive disease associated with defective biliary excretion of copper. The simultaneous occurrence of WD and systemic lupus erythematosus (SLE) has seldom been reported. Therefore, this study aimed to report the co-occurrence of SLE and WD with hepatic involvement in a patient so as to improve the understanding of the coexistence of these two conditions.

**Case presentation:**

A 35-year-old woman with SLE was found to have liver fibrosis during a routinely abdominal ultrasound examination. Her laboratory evaluation showed low serum ceruloplasmin and high 24h urine copper levels. The slit-lamp examination revealed the presence of KayseriFleischer ring in her cornea. Liver biopsy demonstrated the enlargement of the portal area with hyperplasia of the fibrous tissue, infiltration of lymphoid plasma cells, swelling of hepatocytes, and steatosis, demonstrating liver fibrosis. Ensuing genetic testing confirmed the diagnosis of WD.

**Conclusions:**

Clinicians should bear in mind that unexplained liver fibrosis in patients with SLE may be related to WD, so as to avoid a missed or delayed diagnosis.

## Background

Wilsons disease (WD) is an autosomal recessive disease, and is associated with defective biliary excretion of copper. Excessive build-up of copper leads to progressive liver cirrhosis, neurological damage, ophthalmologic manifestations including KayserFleischer (KF) ring, and renal malfunction [1]. WD could occur at any age, but it is mainly observed between 5 and 35years [2]. Among adolescent patients with WD, hepatic symptoms are more common than nervous symptoms. However, the opposite is true in adults [3]. The simultaneous occurrence of WD and SLE has seldom been reported. To improve the understanding of the coexistence of these two conditions, we herein report the co-occurrence of SLE and WD with hepatic involvement in a 35-year-old woman.

### Case presentation

A 35-year-old woman who was affected by recurrent fever and multi-joint pain was diagnosed with SLE in 2015, in accordance with the 1997 American College of Rheumatology (ACR) Classification Criteria [4]. Her symptoms were effectively controlled with methylprednisolone and hydroxychloroquine (HCQ), and she was followed-up regularly at the outpatient department. An annual assessment of important organs in addition to routine examinations (monitoring activity indicators) was performed during the remission period.

During her routine annual health checkup conducted in 2018, the abdominal ultrasound examination unexpectedly revealed a lack of smoothness of the liver surface. She showed no signs of abdominal distension, anorexia, and yellow staining of the skin and mucous membrane. She denied having a history of infectious disease, neurological disturbance, and intermarriage of close relatives. Furthermore, she denied using any medications or components that could have detrimental effects on the liver. Physical examination revealed no abnormalities in the abdomen and nervous system.

Laboratory tests revealed a below-normal albumin level (33g/L; normal range:35.050.0g/L), and serum biochemistry indicated no abnormalities in the hepatic function: total bilirubin (TB), 4.7mol/L, normal range 322mol/L; aspartate aminotransferase (AST), 14 U/L, normal range 1335 U/L; alanine transaminase (ALT), 13.6 U/L, normal range 740 U/L; globulin, 29.6g/L, normal range 2040g/L; international normalized ratio, 1.1, normal range 0.81.5 (Table 1). Moreover, the patient tested negative for a spectrum of autoimmune liver disease antibodies, hepatitis virus, and tumor markers; toxicology screening yielded negative results.

**Table 1:** Serum markers before and after treatment with zinc sulfate for WD and methylprednisolone and HCQ for SLE

Serum markers	Before treatment	After treatment	Reference range
Albumin (g/L)	33	37.9	35.050.0
TB (mol/L)	4.7	8.9	322
AST (U/L)	14	10.6	1335
ALT (U/L)	13.6	12.6	740
Globulin (g/L)	29.6	33.2	2040
INR	1.1	1.3	0.81.5
Ceruloplasmin (g/L)	<0.10	0.150.20	0.20.6

**Fig. 1:**
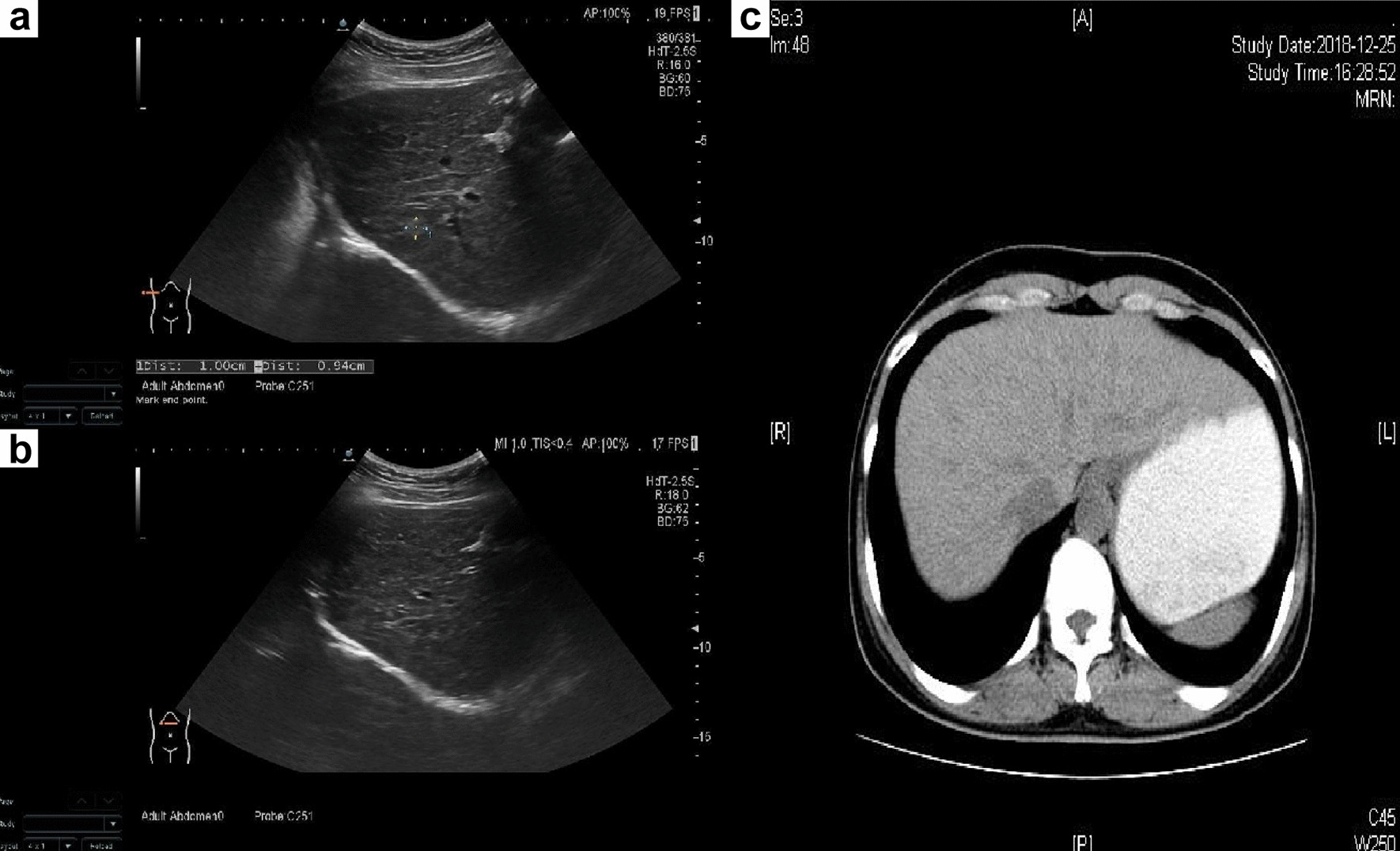
Abodominal ultrasound and CT images of the patient showing early liver cirrhosis. **a**, **b** Abodominal ultrasound revealed unsmooth liver surface, irregular edges, enhanced thickening dot echoes and uneven distribution with several slightly hyperechoic nodules. **c** CT of the upper abdomen showed that the hepatic capsule was wavy

Abodominal ultrasound revealed unsmooth liver surface, irregular edges, enhanced thickening dot echoes and uneven distribution. Several slightly hyperechoic nodules were observed in the liver (Fig.1a, b). CT of the upper abdomen also showed that the hepatic capsule was wavy, indicating early liver cirrhosis (Fig.1c). No ascites were observed. In addition, the presence of venous thromboembolism (VTE) and fatty liver were excluded.

Other tests indicated the presence of leucopenia (2.7810^9^/L) and anemia (hemoglobin level: 112g/L). The patient tested positive for multiple auto-antibodies, including ANA+1:320, anti-dsDNA (++), anti-SSA antibody (+++), anti-Smith antibody (+), anti-Ro52 antibody (+), and anti-rRNP antibody (+++), and showed low levels of serum complement components (C3, C4). The results of urine analysis and renal function tests were within normal limits.

**Fig. 2:**
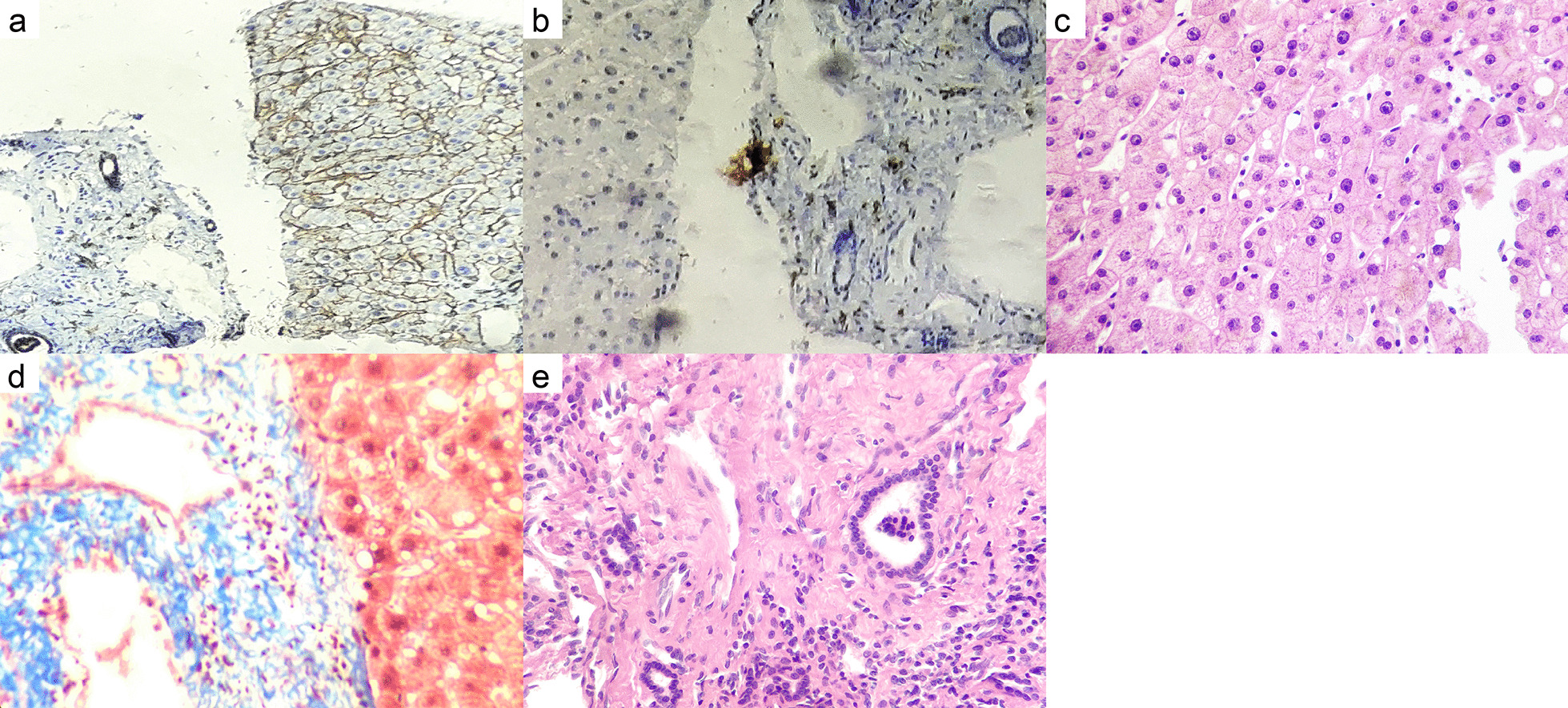
Liver biopsy findings of the 35-year-old female patient. **a** Special staining showed that reticular fiber staining showed the integrity of reticular fiber scaffolds in liver lobules; **b** a small number of plasma cells in the portal area showing the CD38 and CD138 markers; **c** presence of slight swelling, cholesterol, and steatosis of hepatocytes (mixed type of big and small vesicles), hepatocyte nuclei of different sizes, and a small number of bi-nucleate and glycogenic hepatocytes, as shown by HE400; **d** collagen hyperplasia in the portal area, as shown by Masson's staining; **e** widening of the portal area with fibrous tissue proliferation and infiltration of a small number of lymphocytes and plasma cells, as shown by HE100

**Fig. 3:**
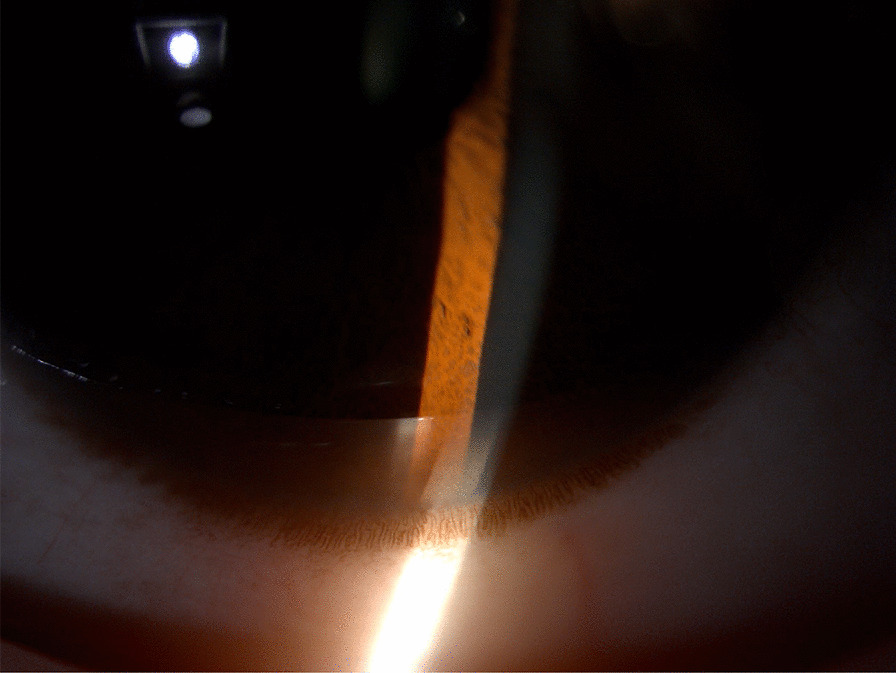
Image obtained during slit lamp examination showing characteristic brown-gold-yellow KayserFleischer (KF) ring at the margin of the cornea and sclera

To determine the cause of liver cirrhosis, liver biopsy, ophthalmic examination, and other metabolic etiology-related tests were conducted. Liver biopsy demonstrated the enlargement of the portal area with hyperplasia of the fibrous tissue, infiltration of lymphoid plasma cells, swelling of hepatocytes, and steatosis (Fig.2). All these pathological findings support the diagnosis of liver fibrosis. Unfortunately, the measurement of the liver copper content could not be conducted because of the presence of limiting laboratory conditions. The slit lamp examination revealed a KF ring in the cornea (Fig.3). The serum ceruloplasmin, serum copper, and 24-h urine copper levels were determined to be<0.10g/L (normal range: 0.20.6g/L), 2.44mol/L (normal range: 11.839.3mol/L) and 110g/24h (normal range:<100g/24h), respectively. The genotype test result revealed a compound heterozygous mutation in the ATP7B gene, which confirmed the presence of disease-causing mutations.

On the basis of the above findings, the patient was definitively diagnosed with WD and SLE. The WD diagnosis was based on the Leipzig scoring system proposed by an international group for the study of WD. The scoring system assessed the patient for hepatic copper content, urinary excretion of copper, serum levels of ceruloplasmin, and neurological symptoms; the presence/absence of KF rings, as well as ATP7B mutations gene that causes copper toxicosis. The score4 points established the diagnosis of WD. The patients Leipzig score was 9 points(KF rings present, score 2; serum ceruloplasmin level<0.1g/L, score 2; urinary copper content 12ULN, score 1; and both chromosomes detected, score 4) [5]. Therefore, the patient was prescribed the complete dosage of zinc sulfate (60mg/time, tid, p.o., 1h before meals) for WD and methylprednisolone and HCQ for SLE. After an 11-month follow-up, abdominal ultrasound examination indicated notable signs of recovery, and the serum ceruloplasmin concentration fluctuated from 0.15g/L to 0.20g/L, which was close to the normal level. Laboratory tests after treatment showed TB of 8.9mol/L, AST of 10.6 U/L, ALT of 12.6 U/L, globulin of 33.2g/L and albumin of 37.9g/L (Table 1).

## Discussion and conclusions

SLE has the potential to affect all organ systems. Hepatic damage is common in SLE; It is usually attributed to non-immune-related diseases (such as viral hepatitis) and adverse effects of drugs (drug-induced damage) [5,7]. In addition, previous studies reported a correlation between SLE-related hepatitis and ribosomal P proteins (anti-ribosomal P) [8, 9]. Previously, the existence of this antibody was thought to be associated with lupus activity and lupus-related psychosis [10]. In addition, studies suggested that lupus hepatitis could occur because of steatosis secondary to corticosteroid treatment. In the current study, the patients medical history and results of tumor marker screening, toxicology screening, and hepatitis virus examination, which were found to be negative, did not support the diagnosis of lupus hepatitis, drug-induced liver damage, and liver fibrosis caused by viral hepatitis.

With regard to the underlying etiology, it was speculated that liver fibrosis in this patient was attributable to an overlapping immune-related syndrome (such as autoimmune hepatitis, AIH) [11, 12]. AIH, which is a chronic inflammatory liver disease, is often associated with the presence of an extra-hepatic immune disease and is prevalent in women. It is usually associated with elevated aminotransferase and -globulin or IgG levels and auto-antibody positivity [13, 14]. Clinically, its manifestations vary significantly from asymptomaticity to acute liver failure. After the exclusion of other chronic liver diseases, the diagnosis of AIH is usually made considering the characteristic serological and histological findings. In this case, although liver biopsy revealed pathological features that are similar to those of AIH, they were nonspecific. Furthermore, AIH-related antibodies have not been detected, was inconsistent with the diagnostic criteria of AIH. Moreover, the etiology of liver fibrosis in the current patient is monistic rather than dualistic as shown by the improvement of liver ultrasound findings after treatment with a zinc-containing drug.

WD is classified as an autosomal recessive genetic disease associated with the pathogenic gene ATP7B. Its mutation can lead to the deterioration or loss of ATPase function, thus leading to a decrease in serum ceruloplasmin synthesis. Furthermore, mutation of this gene contributes to the development of biliary copper excretion disorder and the accumulation of excessive copper in the liver and other organs [15]. In general, symptoms of WD are categorized into hepatic, neurological, mixed, and other types. In teenagers, the incidence of hepatic symptoms is higher than that of other types of symptoms. On the contrary, the incidence of neurological symptoms is higher than that of other types of symptoms in adults. There are many kinds of hepatic manifestations, such as acute liver failure, acute hepatitis, chronic hepatitis, fibrosis, cirrhosis, steatosis, and asymptomatic liver biochemical abnormalities. In addition, biopsy of samples obtained from patients with WD reveals various types of manifestations, including steatosis, ballooning, Mallory bodies, interfacial inflammation, bridging necrosis, and fibrosis; however, none of these manifestations are specific [16]. The clinical manifestations and histological characteristics vary and are complicated, and hence the diagnosis of WD is challenging. Biochemical abnormalities such as abnormal serum ceruloplasmin and 24-h urinary copper excretion levels are important for the diagnosis. Other clinical manifestations include Coombs negative hemolytic anemia and corneal KF ring. In the current study, the patient had asymptomatic fibrosis as the clinical manifestation; furthermore, the patient showed reduced serum ceruloplasmin levels, KF ring, and a complex heterozygote mutation; All these findings conformed to the guidelines for the diagnosis and treatment of WD [17]. Therefore, the diagnosis of WD was confirmed. The patients fibrosis was believed to be attributable to copper deposition. A previous study reported that endemic Tyrolean infantile cirrhosis, a non-Wilsonian hepatic copper toxicosis, is the result of geneenvironment interaction [18]. Environment factors may also be involved in the clinical presentation of WD. However, the present case did not find related environmental factors. It is deserved to be further investigated.

Nevertheless, literature focusing on the coexistence of WD and SLE is scarce. Reportedly, patients with WD who are treated with penicillamine show SLE, which indicates the role of drugs associated with WD and SLE [19]. However, previous study reported that a child diagnosed with WD and treated with penicillamine for 10years before developing nephritis, arthritis, hemolytic anemia, and autoantibodies. Penicillamine-induced lupus was first suspected, and the patient was prescribed oral zinc therapy. However, the symptoms worsened and idiopathic SLE was confirmed [20]. A connection was found between lupus and WD. Santhakumar et al. [21]. accidentally detected the presence of KF rings in the eyes of a 24-year-old female patient with SLE and visual impairment, during an ophthalmic assessment. Additionally, the diagnosis of WD was confirmed on the basis of both serum ceruloplasmin (low) and 24-h urine copper (high) levels. Zhang et al. [22]. also reported the coexistence of SLE and WD in a woman, which exhibited typical neuropsychiatric symptoms. Asymptomatic liver fibrosis as a clinical sign of WD with SLE has not been reported, and an association between SLE and WD has not been clearly understood so far; Therefore, further exploration are needed in this regard.

Due to the relatively frequent multifaceted manifestations of liver diseases in patients with SLE, with an often difficult differential diagnosis each others. Thus, in patients with SLE showing hepatic damage, the possibility of occurrence of metabolic diseases, in addition to drug-induced liver damage, virus-related liver damage, and autoimmune liver disease, should be considered. Early diagnosis and prompt treatment of WD would be beneficial.

## Data Availability

Not applicable.

## Abbreviations

WD: Wilsons disease
SLE: Systemic lupus erythematosus
KF: KayseriFleischer
ACR: American College of Rheumatology
HCQ: Hydroxychloroquine
AIH: Autoimmune hepatitis
CT: Computer tomography
ANA: Antinuclear antibody
IgG: Immunoglobulin G
ULN: Upper limit of normal

## References

[1] Shimizu N, Yamaguchi Y, Aoki T (1999). Treatment and management of Wilsons disease. Pediatr Int.

[2] Steindl P, Ferenci P, Dienes HP, Grimm G, Pabinger I, Madl C (1997). Wilson's disease in patients presenting with liver disease: a diagnostic challenge. Gastroenterology.

[3] Parkinson's Disease and Movement Disorders Study Group, Neurology Branch of Chinese Medical Association. Guidelines for the diagnosis and treatment of hepatolenticular degeneration. Chin J Neurol. 2008;41(8):5669.

[4] Hochberg MC. Updating the American College of Rheumatology revised criteria for the classification of systemic lupus erythematosus. Arthritis Rheum. 1997;40(9):1725. 10.1002/art.1780400928 9324032

[5] Patel S, Beckler MD, Kesselman MM (2019). Lupus and the liver: a case study. Cureus.

[6] Runyon BA, LaBrecque DR, Anuras S (1980). The spectrum of liver disease in systemic lupus erythematosus: report of 33 histologically-proved cases and review of the literature. Am J Med.

[7] De Santis M, Crotti C, Selmi C (2013). Liver abnormalities in connective tissue diseases. Best Pract Res Clin Gastroenterol.

[8] Arnett FC, Reichlin M (1995). Lupus hepatitis: an under-recognized disease feature associated with autoantibodies to ribosomal P. Am J Med.

[9] Carmona-Fernandes D, Santos MJ, Canho H, Fonseca JE (2013). Anti-ribosomal P protein IgG autoantibodies in patients with systemic lupus erythematosus: diagnostic performance and clinical profile. BMC Med.

[10] Bonfa E, Golombek SJ, Kaufman LD, Skelly S, Weissbach H, Brot N (1987). Association between lupus psychosis and antiribosomal P protein antibodies. N Engl J Med.

[11] Beisel C, Weiler-Normann C, Teufel A, Lohse A (2014). Association of autoimmune hepatitis and systemic lupus erythematodes: a case series and review of the literature. World J Gastroenterol.

[12] Choi DH, Kim HK, Park TI, John BM, Kang SH, Lee YS (2008). A case of anti-LKM 1 positive autoimmune hepatitis accompanied by systemic lupus erythematosus. Korean J Gastroenterol.

[13] Leggett BA (1993). The liver in systemic lupus erythematosus. J Gastroenterol Hepatol.

[14] Usta Y, Gurakan F, Akcoren Z, Ozen S (2007). An overlap syndrome involving autoimmune hepatitis and systemic lupus erythematosus in childhood. World J Gastroenterol.

[15] Bull PC, Thomas GR, Rommens JM, Forbes JR, Cox DW (1993). The Wilson disease gene is a putative copper transporting Ptype ATPase similar to the Menkes gene. Nat Genet.

[16] Liang X, Yang R, Wu Z, Wang N, Li X, Wang X. Guidelines for the dignosis and treatment of wilson's disease. In: Collected papers from the 11th national conference of neurology. Changchun, China. Neurology Branch of Chinese Medical Association. 2008.

[17] European Association for the Study of the Liver (2012). EASL clinical practice guidelines: Wilsons disease. J Hepatol.

[18] Mller T, Feichtinger H, Berger H, Mller W (1996). Endemic Tyrolean infantile cirrhosis: an ecogenetic disorder. Lancet.

[19] Walshe J (1981). Penicillamine and the SLE syndrome. J Rheumatol Suppl.

[20] Dell'era L, Boati E, Nebbia G, Corona F (2012). Wilson's disease treated with penicillamine and lupus erythematosus: related or distinct entities?. Minerva Pediatr.

[21] Santhakumar R, Gayathri K, Ramalingam P, Manjunath B, Karuppusamy N, Vetriveeran B (2016). Wilson's disease with systemic lupus erythematosus. J Assoc Phys India.

[22] Zhang Y, Wang D, Wei W, Zeng X (2018). Wilsons disease combined with systemic lupus erythematosus: a case report and literature review. BMC Neurol.

